# Digital Device in Postextraction Implantology: A Clinical Case Presentation

**DOI:** 10.1155/2014/327368

**Published:** 2014-12-29

**Authors:** A. E. Borgonovo, F. Rigaldo, D. Battaglia, D. Re, A. B. Giannì

**Affiliations:** ^1^School of Oral Surgery, Istituto Stomatologico Italiano, University of Milan, Milan, Italy; ^2^Department of Oral Rehabilitation, Istituto Stomatologico Italiano, Milan, Italy; ^3^Department of Maxillofacial Surgery, Fondazione IRCCS Ospedale Maggiore Policlinico, University of Milan, Milan, Italy

## Abstract

*Aim*. The aim of this work is to describe a case of immediate implant placement after extraction of the upper right first premolar, with the use of CAD/CAM technology, which allows an early digital impression of the implant site with an intraoral scanner (MHT 3D Progress, Verona, Italy). *Case Report*. A 46-year-old female was referred with a disorder caused by continuous debonding of the prosthetic crown on the upper right first premolar. Clinically, there were no signs, and the evaluation of the periapical radiograph showed a fracture of the root, with a mesial well-defined lesion of the hard tissue of the upper right first premolar, as the radiolucent area affected the root surface of the tooth. It was decided, in accordance with the patient, that the tooth would be extracted and the implant (Primer, Edierre implant system, Genoa, Italy) with diameter of 4.2 mm and length of 13 mm would be inserted. After the insertion of the implant, it was screwed to the scan abutment, and a scan was taken using an intraoral scanner (MHT 3D Progress, Verona, Italy). The scanned images were processed with CAD/CAM software (Exocad DentalCAD, Darmstadt, Germany) and the temporary crown was digitally drawn (Dental Knowledge, Milan, Italy) and then sent to the milling machine for production with a composite monoblock. After 4 months, when the implant was osteointegrated, it was not necessary to take another dental impression, and the definitive crown could be screwed in. *Conclusion*. The CAD/CAM technology is especially helpful in postextraction implant for aesthetic rehabilitation, as it is possible to immediately fix a provisional crown with an anatomic shape that allows an optimal healing process of the tissues. Moreover, the removal of healing abutments, and the use of impression copings, impression materials, and dental stone became unnecessary, enabling the reduction of the chair time, component cost, and patient's discomfort. However, it is still necessary for scientific research to continue to carry out studies on this procedure, in order to improve the accuracy, the reliability, and the reproducibility of the results.

## 1. Introduction

Computer aided design (CAD) and computer aided manufacturing (CAM) have been widely used by dental health professionals for over twenty-five years, and this technique has evolved over the last three decades.

In 1971 Duret et al. introduced CAD/CAM in restorative dentistry [1] and in 1983 the first dental CAD/CAM restoration was manufactured [2].

This technology, which is used in both the dental laboratory and the dental office, can be applied for dental restoration using prefabricated ceramic monoblocks, such as inlays, onlays, veneers, crowns, and fixed partial dentures [3].

The goal of treatment in modern implant dentistry has shifted from recovering masticatory function to recovering both the aesthetics and the function of the lost tooth.

Also thanks to the introduction of CAD/CAM technology, it is now possible for the prosthetic-implant restoration to be in harmony with the surrounding dentition [4, 5]. Indeed CAD/CAM technology allows the clinician to design improved configuration for each individual case, creating an anatomically ideal abutment.

With this abutment, the clinician can control the emergence profile, which is essential for long-term aesthetics and at times compensates for the fixture position to occlusal arch discrepancies [6, 7].

Moreover, the CAD/CAM technique eliminates further potential dimensional inaccuracies inherent to casting and waxing, which enables precise reproduction of the intended prosthetic design [8].

The aim of this paper is to describe a case of immediate implant placement after upper right first premolar extraction, with the use of CAD/CAM technology, which allows an early digital impression of the implant site with intraoral scanner (MHT 3D Progress, Verona, Italy).

Furthermore, thanks to digital implant libraries, CAD modeling, and CAM preprocessing of Dental Knowledge, it was possible to manufacture with a milling machine a temporary crown that was immediately positioned on the implant. This technique permitted the patient to leave the dental office with provisional rehabilitation.

## 2. Case Report

A 46-year-old female was referred to the Department of Oral Rehabilitation of the Istituto Stomatologico Italiano, University of Milan, Italy, with a disorder caused by continuous debonding of the prosthetic crown on the upper right first premolar.

**Figure 1:**
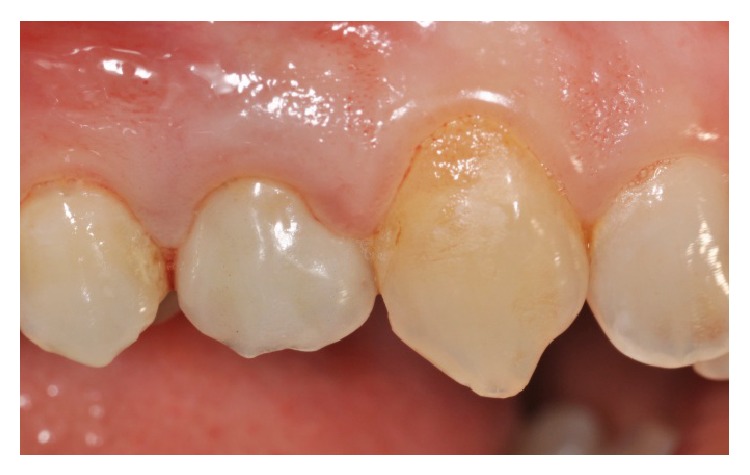
Vestibular view of the prosthetic crown on the upper right first premolar.

**Figure 2:**
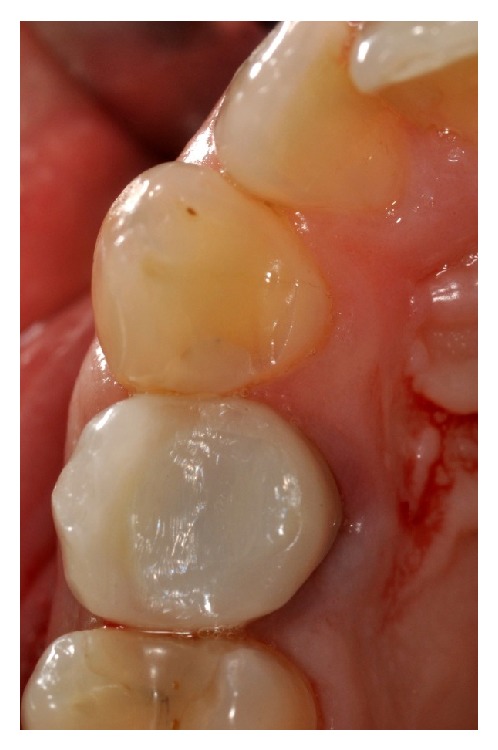
Occlusal view of the prosthetic crown on the upper right first premolar.

**Figure 3:**
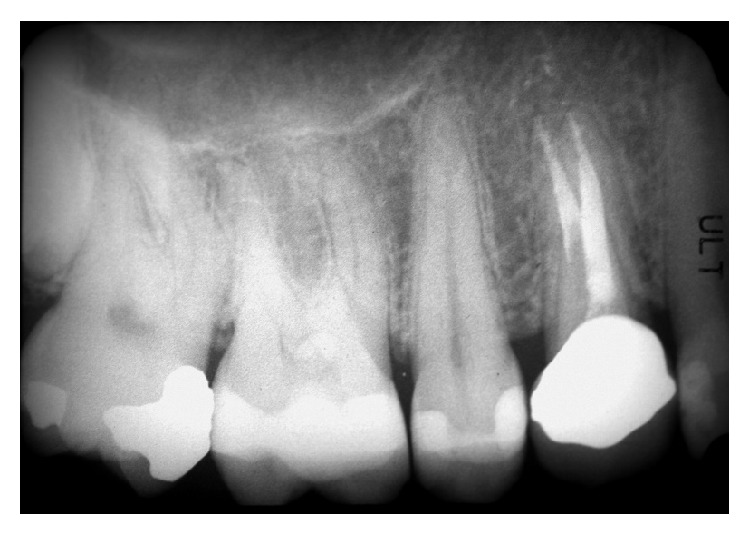
Periapical radiograph showed a fracture of the root, with a mesial well-defined lesion of the hard tissue of the upper right first premolar, as the radiolucent area affected the root surface of the tooth.

Clinically, there were no signs (Figures 1 and 2), and the evaluation of the periapical radiograph showed a fracture of the root, with a mesial well-defined lesion of the hard tissue of the upper right first premolar, as the radiolucent area affected the root surface of the tooth (Figure 3).

**Figure 4:**
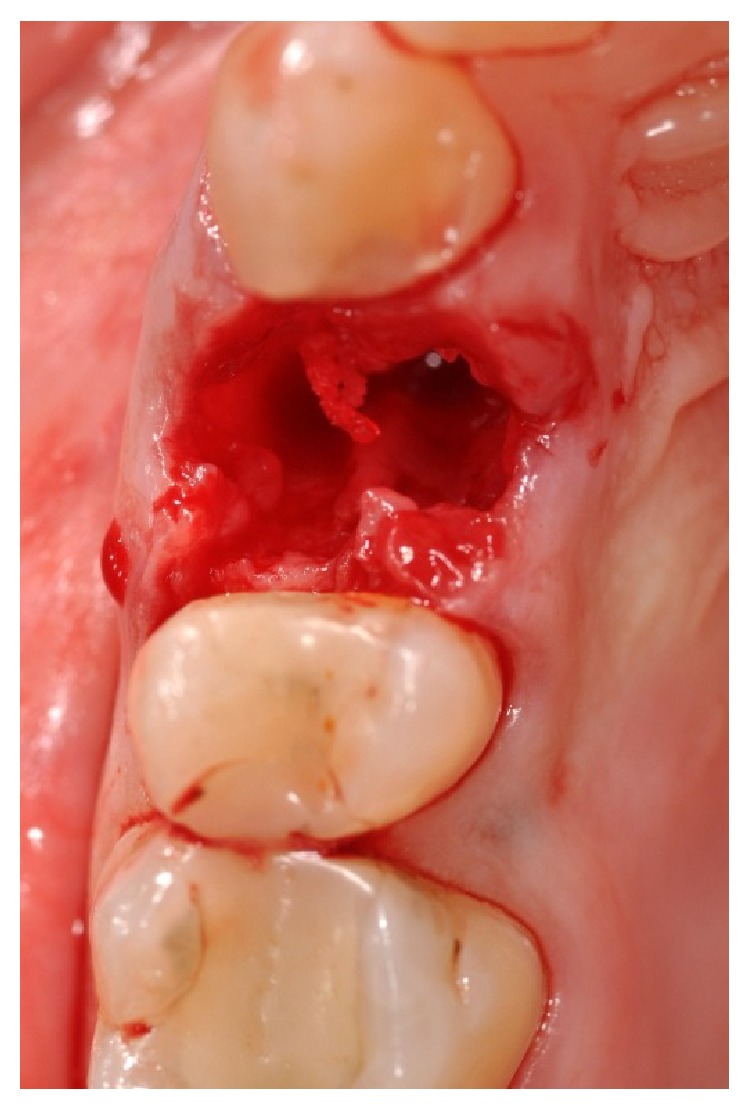
Upper right first premolar area after the extraction of the tooth.

**Figure 5:**
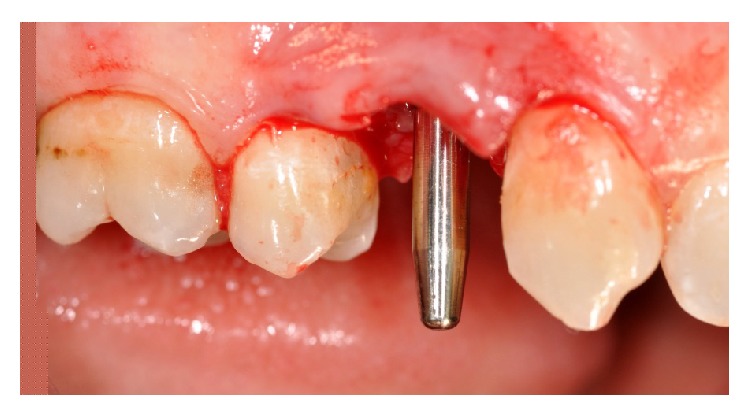


**Figure 6:**
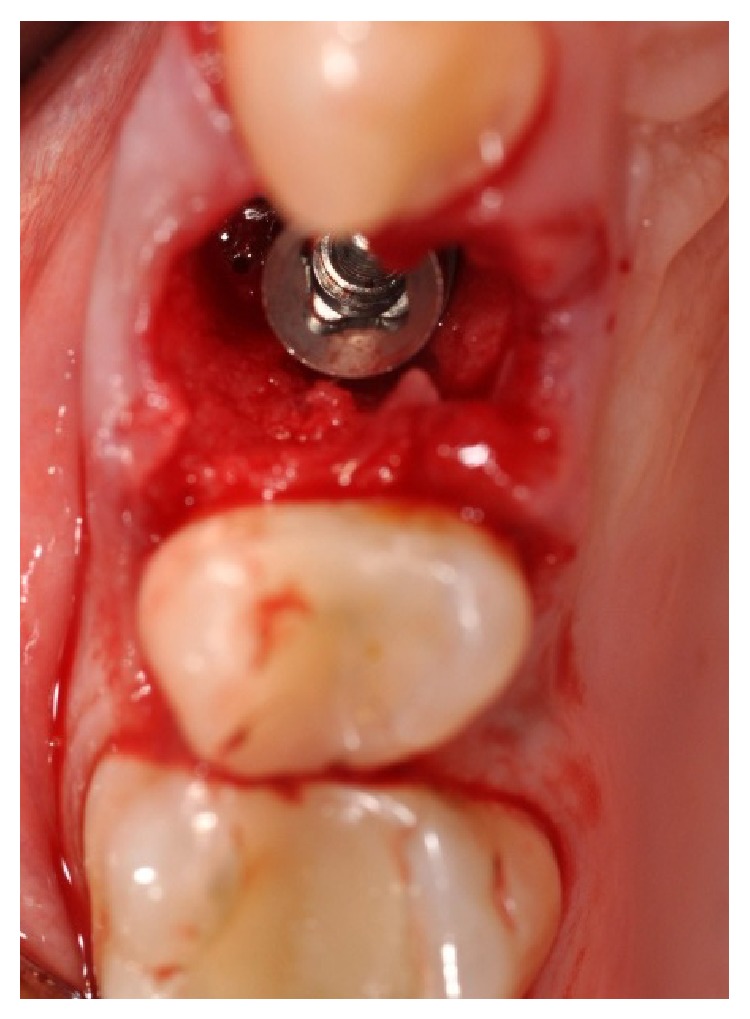
Early placement of the implant.

In accordance with the patient, it was decided that the tooth would be extracted and the implant (Primer, Edierre implant system, Genoa, Italy) with diameter of 4.2 mm and length of 13 mm would be inserted (Figures 4, 5, and 6).

Seven days before surgery, the patient underwent professional oral hygiene and she was instructed to start rinsing her mouth twice a day with chlorhexidine 0.2% (Corsodyl, Glaxo, UK) for two weeks after surgery. Antibiotic prophylaxis with 2 gr of amoxicillin and clavulanic acid (Laboratori Eurogenerici, Milan, Italy) was prescribed one hour prior to surgery. This therapeutic protocol was made for the aesthetic position of the tooth extracted.

The criteria for immediate implant treatment success are traumatic tooth extraction, sterilization, and a minimally invasive surgical approach, as well as implant primary stability [9–11]. The durability of postextraction implants is high and is comparable to the durability of implants placed in healing sites [12].

During the surgical placement of the prosthetic driven implant, it was necessary to execute a transplant with an organic bovine bone (Bio-Oss 0.5 g, Geistlich Pharma AC, Wolhusen, Switzerland) and autogenous bone chips, due to a marginal defect between the implant surface and the inner wall of the extraction socket which exceeded 2 mm [13]. The flap was released through periosteal incisions to attain primary wound closure and was saturated with 4/0 monofilament suture (Premilene, Braun Melsungen, Germany).

**Figure 7:**
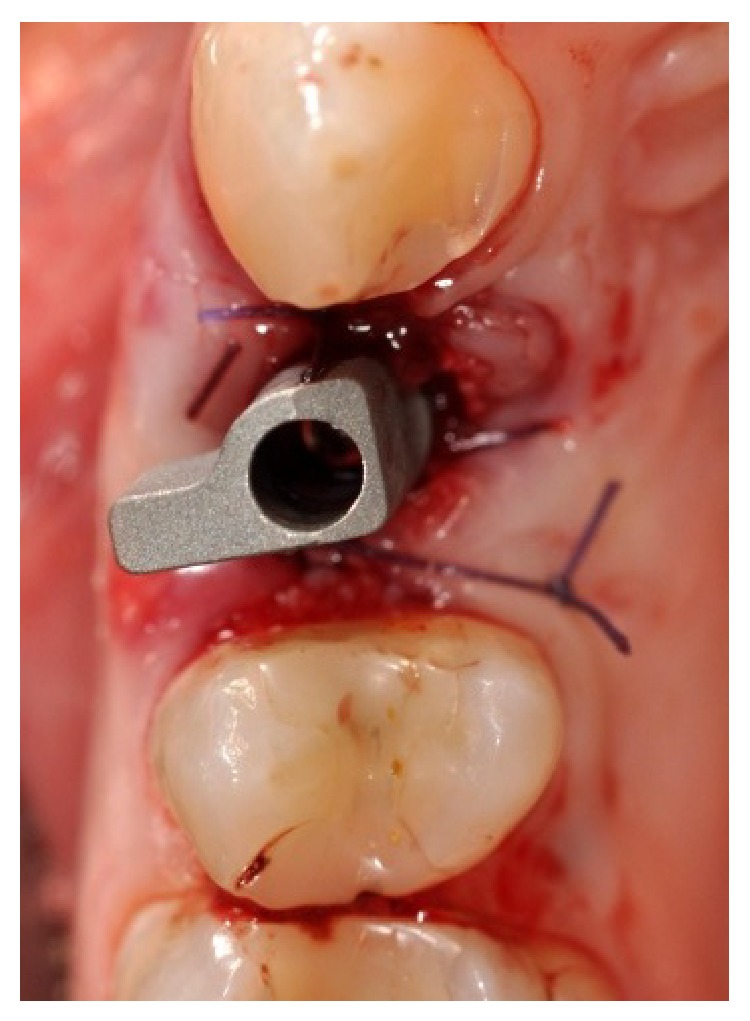
Screwing of the scan abutment, needful to take a scanning with MHT* 3D Progress* intraoral scanner. Occlusal view.

Immediately after implant insertion, an X-ray picture was taken in order to verify the correct implant position (Figure 7).

**Figure 8:**
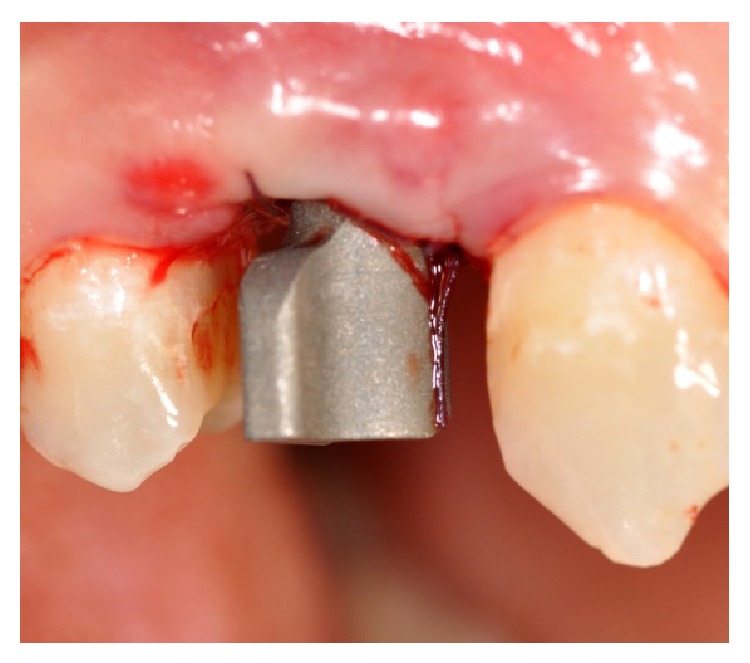
Screwing of the scan abutment, needful to take a scanning with MHT* 3D Progress* intraoral scanner. Vestibular view.

After the insertion of the implant, it was screwed to the scan abutment (Figures 7 and 8), so that a scan could be taken with an intraoral scanner (MHT 3D Progress, Verona, Italy).

Apart from the scan abutment on all sides (distal, mesial, buccal, palatal, and occlusal), it was also necessary to scan the closed teeth on all sides, including the teeth of opposing arches, and the buccal side of the upper and lower teeth during the occlusion.

**Figure 9:**
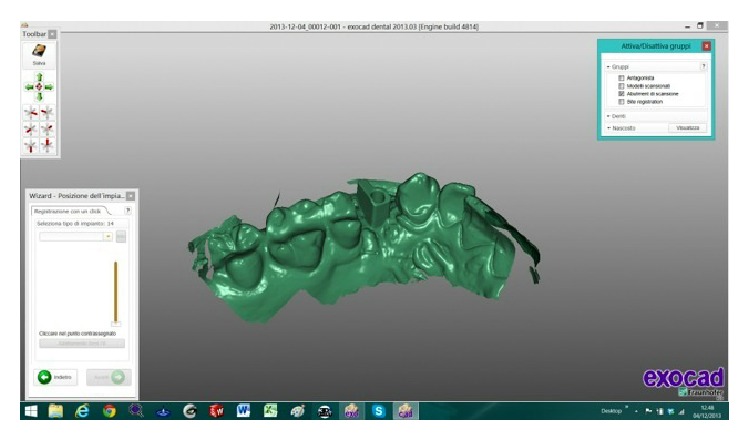


**Figure 10:**
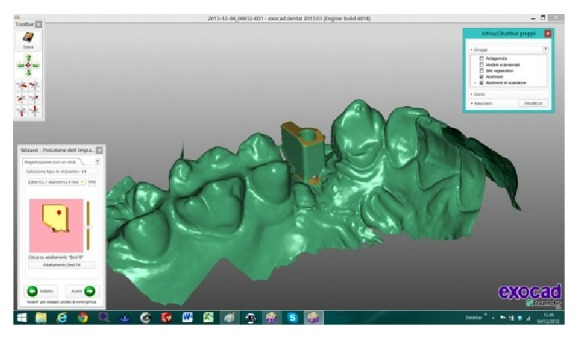
The scanned images were processed with Exocad DentalCAD platform.

**Figure 11:**
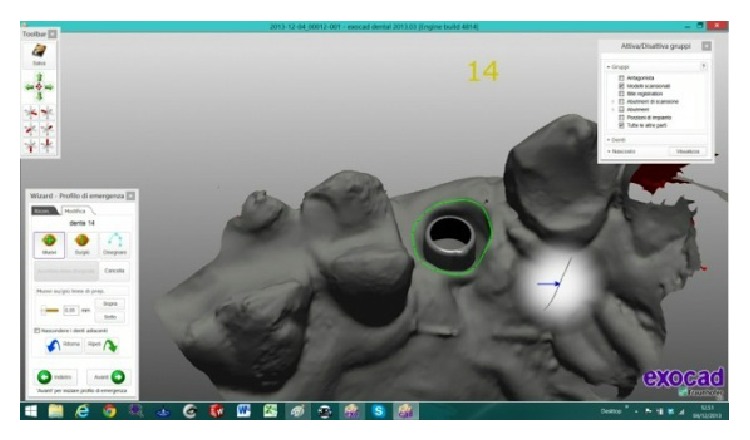


**Figure 12:**
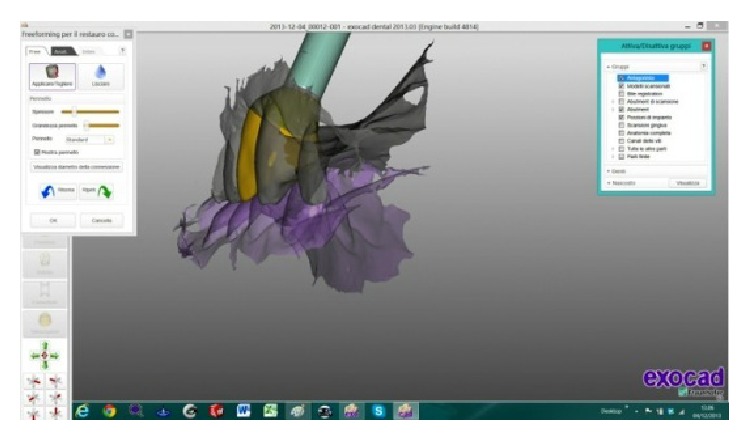
The temporary crown was digitally drawn before sending to the 3D printer.

The scanned images were processed by Dental Knowledge, Milan, Italy, with CAD/CAM software (Exocad DentalCAD, Darmstadt, Germany) (Figures 9 and 10) and the temporary crown was digitally drawn (Figures 11 and 12) and then sent to the milling machine for production with a composite monoblock (Dental Knowledge, Milan, Italy).

**Figure 13:**
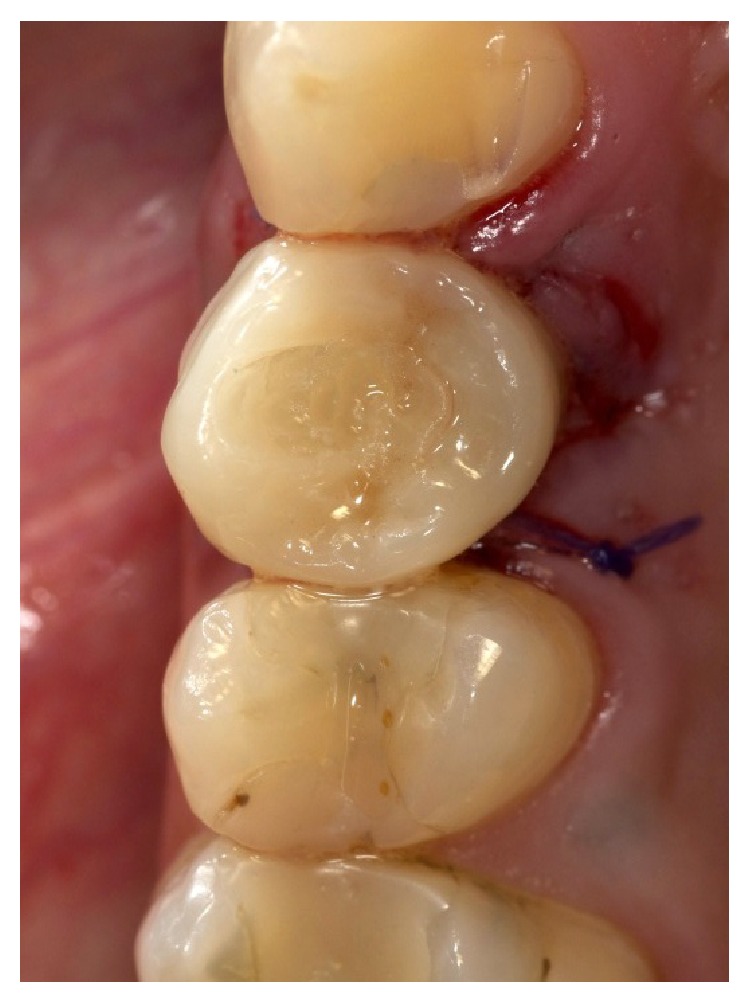
The provisional rehabilitation was immediately fixed removing maximum intercuspidation contacts to ensure the osteointegration.

The provisional rehabilitation was immediately fixed removing maximum intercuspidation contacts to ensure the osteointegration (Figure 13).

After 4 months, when the implant was osteointegrated, without the necessity to take another dental impression, it was possible to digitally draw the custom titanium framework with an anatomic shape and a shoulder placed around the abutment head, which hid the titanium.

**Figure 14:**
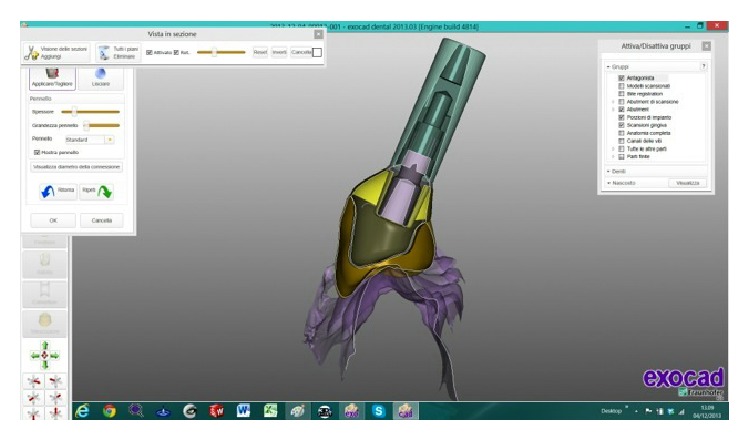
The definitive prosthetic crown was digitally drawn with Exocad DentalCAD platform.

**Figure 15:**
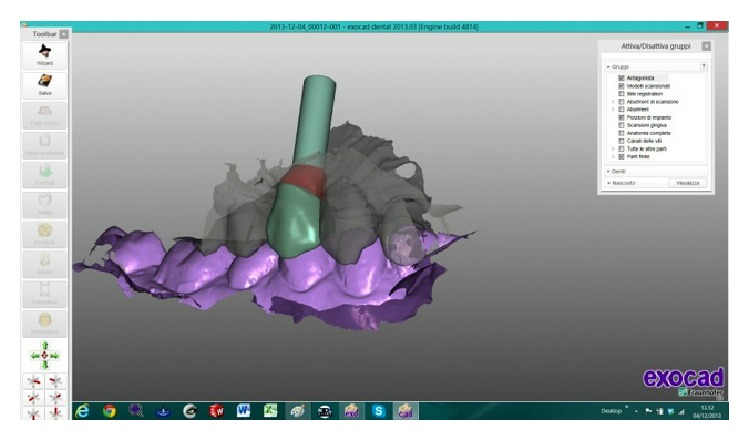
It was possible to digitally draw the custom titanium framework with an anatomic shape and a shoulder placed around the abutment head.

**Figure 16:**
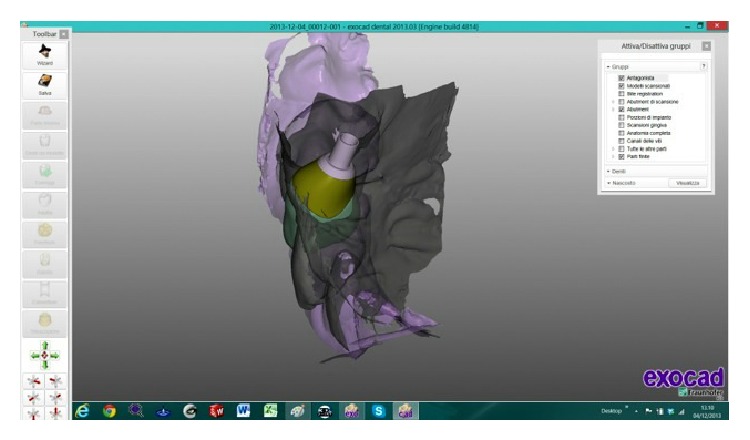
The custom titanium framework with an anatomic shape and a shoulder placed around the abutment head was hidden by the feldspathic porcelain for aesthetics and definitive shape.

**Figure 17:**
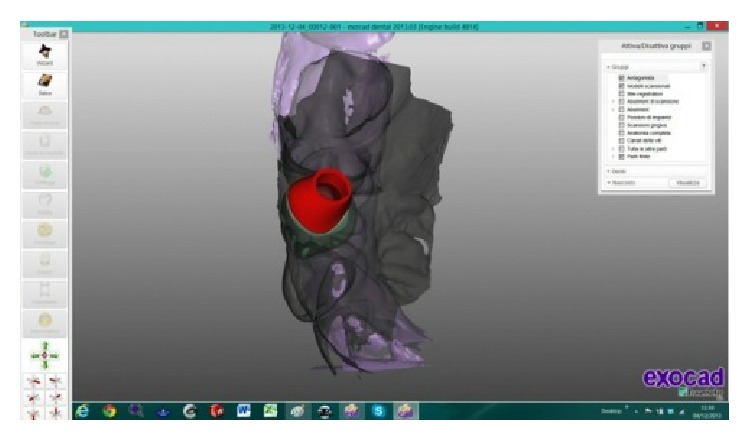


**Figure 18:**
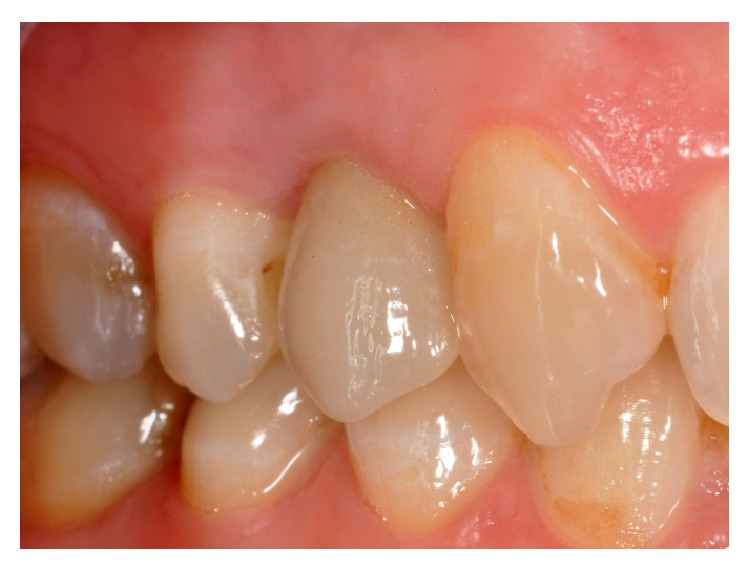
Screwing of the definitive crown. Vestibular view.

**Figure 19:**
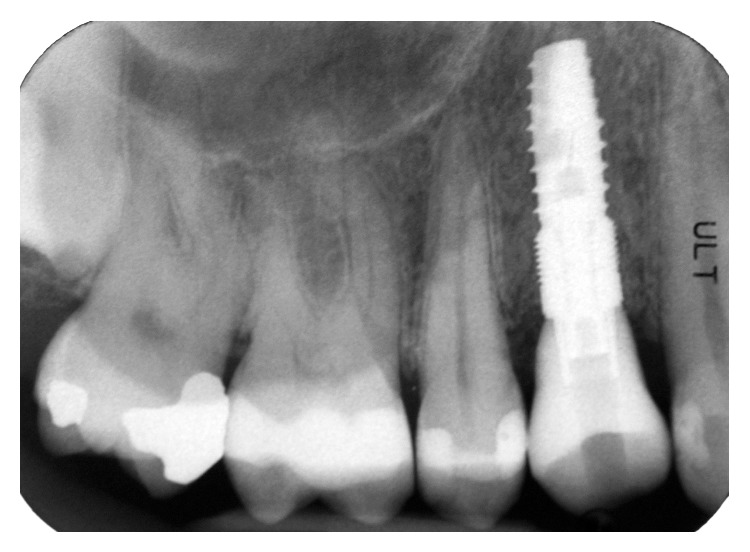
Radiographic view of the implant with the definitive crown screwed.

After it was digitally designed (Figures 14, 15, 16, and 17) the framework was veneered with feldspathic porcelain for aesthetics and definitive shape and it was possible to screw the definitive crown (Figures 18 and 19).

## 3. Discussion

The successful rehabilitation with implant-supported fixed restorations in the aesthetic zone remains one of the biggest challenges in implant dentistry [14, 15]; for this reason the scientific research in aesthetic dentistry is in continuous development.

The digital workflow allows the manufacturing of customized abutments and customized crowns with ideal soft tissue maintenance in combination with high-performance restoration material [16].

The use of the intraoral scanner (MHT 3D Progress, Verona, Italy) and of the digital libraries of Dental Knowledge enables the acquisition of digital data showing the approximate tooth size (10 × 10 × 18 mm) in just 1/10 of a second, by directly reading the tissues morphology.

The advantages of the digital impression technique are that the acquired data is shown in real time on the PC screen and can be examined without touching the computer or just by using the mouse.

Moreover, it eliminates the need for the use of dental impression materials and dental stone, thus potentially providing greater accuracy of final restorations by avoiding dimensional stability problems relating to those materials.

This technique may also reduce the cost of components, as there is no need for impression copings or implant analogs. It may also be considered more comfortable for the patients, as it eliminates the impression procedures that might activate a gag reflex.

In addition, it may save chair time as the final restorations can be fabricated faster than when using a conventional impression technique with impression materials and stone cast. The technique consists of only two sessions, one for the first scanning and one for the insertion of the restorations [17].

With a milling machine installed in the dental clinic the restoration could be installed within 2 hours after the implant insertion.

Most of the digital systems on the market use scanning powder, but the thickness of the scanning powder is not easy to control, thus compromising the precision of the scans [18]. The intraoral scanner (MHT 3D Progress, Verona, Italy) uses a powderless digital impression without it reducing the accuracy.

In addition to the many advantages, this technique also presents some disadvantages, including the need for additional training and experience, lack of space for the scanner tip due to the morphology of the scanner being cumbersome in the posterior region, scanning difficulty, challenge for clinician caused by the saliva flow and patient movement, and finally the high start-up cost of the scanners which may limit their use [19].

## 4. Conclusion

CAD/CAM technology applied to implant surgery allows the production of high resistance and high density crowns and also the manufacture of implant abutments and surgical guides.

This technology is especially helpful in postextraction implant for aesthetic rehabilitation, as it is possible to immediately fix a provisional crown with an anatomic shape that allows an optimal healing process of the tissues.

With the presented technique, the removal of healing abutments and the use of impression copings, impression materials, and dental stone became unnecessary, enabling the reduction of the chair time, component cost, and patient's discomfort.

However, it is still necessary that the scientific research increases the number of studies on this procedure, in order to improve the accuracy, the reliability, and the reproducibility of the results.
